# The Xp10 Bacteriophage Protein P7 Inhibits Transcription by the Major and Major Variant Forms of the Host RNA Polymerase via a Common Mechanism

**DOI:** 10.1016/j.jmb.2016.08.004

**Published:** 2016-10-09

**Authors:** D.R. Brown, C.M. Sheppard, L. Burchell, S. Matthews, S. Wigneshweraraj

**Affiliations:** MRC Centre for Molecular Microbiology and Infection, Imperial College London, SW7 2AZ, UK

**Keywords:** RNAP, RNA polymerase, RPc, closed promoter complex, RPo, open promoter complex, β′ NTD, amino-terminal domain of the β′ subunit, CBD, core binding domain EMSA, ​Electrophoretic Mobility Shift Assay, RNA polymerase, σ factor, bacteriophage, transcription regulation, bacteria

## Abstract

The σ factor is a functionally obligatory subunit of the bacterial transcription machinery, the RNA polymerase. Bacteriophage-encoded small proteins that either modulate or inhibit the bacterial RNAP to allow the temporal regulation of bacteriophage gene expression often target the activity of the major bacterial σ factor, σ^70^. Previously, we showed that during *Xanthomonas oryzae* phage Xp10 infection, the phage protein P7 inhibits the host RNAP by preventing the productive engagement with the promoter and simultaneously displaces the σ^70^ factor from the RNAP. In this study, we demonstrate that P7 also inhibits the productive engagement of the bacterial RNAP containing the major variant bacterial σ factor, σ^54^, with its cognate promoter. The results suggest for the first time that the major variant form of the host RNAP can also be targeted by bacteriophage-encoded transcription regulatory proteins. Since the major and major variant σ factor interacting surfaces in the RNAP substantially overlap, but different regions of σ^70^ and σ^54^ are used for binding to the RNAP, our results further underscore the importance of the σ–RNAP interface in bacterial RNAP function and regulation and potentially for intervention by antibacterials.

## Highlights

• Xp10 phage transcription regulator P7 inhibits transcription by RNAP containing σ^54^.
• P7 prevents the productive engagement of the σ^54^–RNAP with the promoter DNA.
  • P7 disrupts preformed σ^54^–RNAP-promoter complexes.

## Graphical abstract

Graphical abstract

**Figure:**
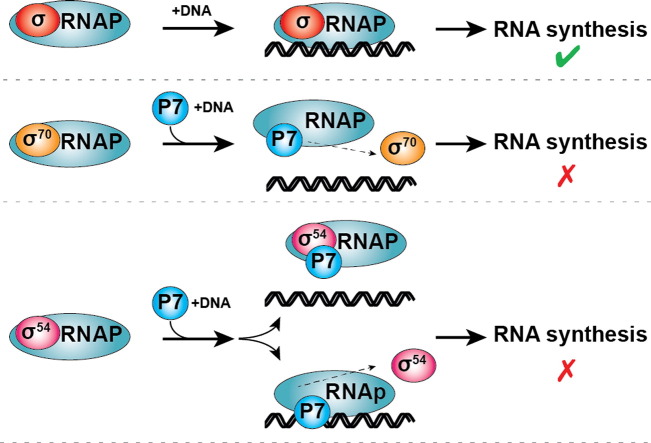

Central to the regulation of bacterial gene expression is the bacterial RNA polymerase (RNAP), which is a complex multisubunit enzyme responsible for the transcription of RNA from the DNA template. The catalytic “core” of the RNAP is composed of five subunits α_2_ββ′ω (E) and is reliant upon the binding of a dissociable sigma (σ) factor subunit for “holoenzyme” (α_2_ββ′ωσ; Eσ) formation and promoter-specific initiation of transcription (reviewed in Ref. [1]). All bacteria have at least one essential major σ factor that serves to transcribe genes required for cell viability and a varying number of alternate σ factors for the execution of specific transcriptional programs. *Escherichia coli*, for example, encodes six alternate σ factors in addition to the major σ^70^ factor (reviewed in Ref. [2]). Transcription initiation at a prototypical σ^70^-dependent housekeeping promoter initially involves the engagement of the Eσ^70^ with conserved hexanucleotide sequences of the promoter, which are located at positions − 35 and − 10 with respect to the transcription initiation site at + 1, and results in the formation of a short-lived Eσ^70^–promoter complex (RPc). The isomerization of the RPc to the transcriptionally proficient promoter complex (RPo) is accompanied by large-scale conformational rearrangements in both the DNA and the RNAP, primarily in the β, β′ and σ^70^ subunits. In the RPo, the DNA duplex is locally melted and the + 1 site on the template strand is positioned at the catalytic centre of the RNAP; the double-stranded DNA, which is downstream of the + 1 site, is cradled in the downstream DNA binding channel that consists of a trough formed by the β′ jaw, β downstream lobe, β′ clamp, and β′ region G non-conserved domain (GNCD) (reviewed in Ref. [3]). The different interfaces between the σ^70^ factor and the RNAP in the holoenzyme, RPc, and RPo, and the transition between these states are extensive, dynamic, and functionally specialised [4], [5], [6], [7]. In *E. coli*, all alternate σ factors (except σ^54^) belong to the major σ^70^ class and share three regions of conserved sequences [regions 2–4, with the exception of extracytoplasmic function (ECF) σ factors that do not contain region 3]. Subregions 2.4 and 4.2 of regions 2 and 4 of *E. coli* σ^70^ are responsible for the recognition of the conserved − 10 and − 35 double-stranded promoter sequences, respectively [2], [5], [6]. In the holoenzyme, subregion 2.2 of σ^70^ makes extensive contact to the β′ clamp helices, which comprise of a coiled-coil motif and constitutes the major σ docking site in the RNAP. Region 4 makes extensive interactions with β flap domain and the conserved features (notably the β′ zipper and β′ zinc binding domain) in the amino-terminal domain of the β′ subunit (hereafter called β′ NTD) [7]. The interactions between region 4 of σ^70^ and the β and β′ subunit are important for the binding of the holoenzyme to conserved − 35 promoter sequence and during promoter clearance for the appropriate exiting of the nascent RNA from the RNAP [5], [6], [8], [9].

Regulating the activity of the RNAP is a key mechanism in controlling gene expression and is often orchestrated by transcription regulators that interact with the RNAP to modulate its activity. Therefore, the RNAP often serves as a nexus for interaction of transcription regulators to fine-tune gene expression to match cellular requirements. Unsurprisingly, some bacteriophages (phages) have evolved strategies to alter the activity of host RNAP during infection to allow the temporal and coordinated usage of the host and phage RNAP for phage gene expression [10]. This modulation can occur in two ways, either through covalent modifications, such as phosphorylation or ADP ribosylation, of target sites on the RNAP or through the binding of low-molecular-weight, phage-encoded proteins [11]. Many phage-encoded host transcription regulators interfere with host RNAP activity by modulating the σ factor–RNAP interface during transcription initiation. For example, the T7 phage protein Gp2 binds in the downstream DNA binding channel and prevents the obligatory displacement of the amino-terminal domain of σ^70^ from the downstream DNA binding channel to allow RPo formation [12], [13]. The T4 phage protein AsiA binds to the region 4 of σ^70^ and structurally remodels it [14]. Consequently, σ^70^ region 4 can no longer bind to the conserved − 35 promoter sequence of host promoters and to the β flap domain of the RNAP. This, in turn, allows another T4 protein, MotA, to interact with the far carboxyl terminal region of σ^70^ and divert the host RNAP from host promoters to T4 phage middle gene promoters, which do not contain conserved − 35 promoter elements [15]. Recently, we demonstrated that a protein called P7, which is expressed by the *Xanthomonas orzyae* infecting Xp10 phage, inhibits the host RNAP by causing the displacement of the σ^70^ during RPc formation [16]. The interface between P7 and the RNAP is complex and involves three different subunits: P7 first docks onto the β′ NTD and positions itself proximal to the β flap domain. Subsequently, a new interaction surface is unveiled on P7 that interfaces with the tip helix of the β flap, thereby altering the interface between σ^70^ region 4 and the β flap. Thus, upon engagement with the promoter DNA, the σ^70^ factor becomes displaced from the RNAP, which consequently prevents the formation of the RPc [16], [17]. P7 also interacts with the ω subunit of the host RNAP; however, this interaction seems to be dispensable for its role as a transcription initiation inhibitor [18].

σ^54^, which is present in many bacterial species, is the major variant bacterial σ factor and is unrelated to the σ^70^ family in sequence, structure, function, and regulation (reviewed in Refs [19], [20]). Contrasting the scenario at prototypical σ^70^-dependent promoters and at σ^54^-dependent promoters, the Eσ^54^ forms an RPc that requires conformational remodelling by a specialised type of activator ATPase for conversion into a transcriptionally proficient RPo. The comparison of the Eσ^70^ and Eσ^54^ structures reveals that, overall, both σ factors occupy overlapping positions in the RNAP [21]. In the case of Eσ^70^, the region 4 of σ^70^ interacts with the β flap and β′ NTD domain, respectively. In Eσ^54^, a region comprising amino acids 120–250, called the “core binding domain” (CBD), which is obligatory for the docking of σ^54^ to the RNAP, makes extensive contacts to the β′ NTD and the β flap domain (Fig. 1). In other words, in Eσ^54^, the P7 and the CBD bind to substantially overlapping surfaces of the RNAP β and β′ subunits (Fig. 1), and therefore, in this study, we investigated the effect of P7 on Eσ^54^-dependent transcription.

Residues 6–9 Asparagine, Leucine, Phenylalanine, Asparagine (NLFN) of the β′ subunit of *X. oryzae* RNAP are the major determinants for P7 binding [22]. Since the *E. coli* RNAP contains different amino acids at this position Lysine, Phenylalanine, Leucine and Asparagine (KFLN) and is therefore resistant to inhibition by P7, we previously constructed a P7-sensitive version of the *E. coli* RNAP by replacing 6–9 aa of the *E. coli* β′ subunit with the corresponding residues of the *X. oryzae* RNAP to study the effect of P7 on σ^70^-dependent transcription [16]. We conducted an *in vitro* transcription assay using the well-characterised *Sinorhizobium meliloti nifH* promoter and the catalytic domain of the *E. coli* Phage shock protein F (PspF_1–275_) [23] to determine the effect of P7 on ^P7S^Eσ^54^ activity. Results revealed that the amount of the UpGpGpG transcript synthesised from *S. meliloti nifH* promoter by ^P7S^Eσ^54^ was substantially reduced (by ~ 80%) in the presence of just an equimolar amount of P7 to ^P7S^Eσ^54^ [Fig. 2a (i), lane 2]. A similar effect of P7 on ^P7S^Eσ^54^ activity was observed in *in vitro* transcription reactions with two different σ^54^-dependent promoters, *E. coli glnHp*2 and *relAp4* promoters [Fig. 2a (ii) and (iii), respectively]. As expected, control reactions with the ^WT^Eσ^54^ confirmed that the observed reduction in the activity of ^P7S^Eσ^54^ at all three σ^54^-dependent promoters was specific to P7 [Fig. 2a (i–iii), lanes 5 and 6]. We next investigated the step at which P7 exerts its inhibitory effect on transcription initiation by ^P7S^Eσ^54^ by adding P7 to different steps of the *in vitro* transcription reaction (Fig. 2b, schematic). The results showed that the activity of ^P7S^Eσ^54^ was reduced by ~ 90% when approximately fourfold molar excess P7 was either added to the core RNAP prior to holoenzyme formation or to the preformed holoenzyme prior to RPc formation (Fig. 2b, lanes 2 and 3). However, when P7 was added to the RPc and to the RPo, the inhibitory effect of P7 on ^P7S^Eσ^54^ was reduced and ^P7S^Eσ^54^ retained ~ 40–60% activity compared to the reaction where no P7 was present (Fig. 2b, lanes 4 and 5). Thus, it seems that P7 is able to adversely affect the transcriptional activity of ^P7S^Eσ^54^ at all stages during transcription initiation with the maximum inhibitory effect exerted prior to RPc formation. In contrast, P7 can fully abolish the activity of ^P7S^Eσ^70^ on the *lac*UV5 promoter at any point prior to RPo formation; however, once the RPo is formed, P7 has no detectable effect on the amount of ApApUpU transcript synthesised by ^P7S^Eσ^70^ from the *lac*UV5 promoter (Fig. 2c). We thus considered whether P7 could have any adverse effects on the activity of the activator ATPase *per se*. To rule out this possibility, we conducted a simple Electrophoretic Mobility Shift Assay (EMSA)-based assay to monitor the ability of the activator ATPase to remodel a σ^54^-promoter complex (which results in a super-shifted σ^54^-promoter complex; ssσ^54^–^32^P–*nifH* in Fig. 2d) in the presence of P7 [24]. Results shown in Fig. 2d indicate that P7 did not have any detectable, adverse effect on the activity of the activator ATPase. Thus, the results so far suggest that at σ^54^-dependent promoters, P7 does not interfere with the activity of the activator ATPase, inhibits a step(s) *en route* to RPo, and can still, to a certain degree, interact with and adversely affect the RPo once it has formed.

To identify the mechanism by which P7 inhibits Eσ^54^ activity, we conducted EMSAs with ^32^P-labelled *nifH* promoter probe to determine if P7, like at σ^70^-dependent promoters, inhibits transcription initiation by preventing RPc formation by Eσ^54^. As shown in Fig. 3a, the wild-type and P7-sensitive core RNAP (in the absence of σ^54^) migrate as two complexes (C1 and C2) under our conditions (lanes 4 and 13). We note that the C1 complex is more prominent in the reaction containing the wild-type core RNAP than it is in the reaction with P7-sensitive core RNAP, and we suggest that this possibly indicates conformational differences between the two enzymes. In the presence of σ^54^, the C1 complex disappeared, and a third complex, C3, appeared (Fig. 3a, lanes 6 and 15). However, the C2 complex remains, although to a much lesser extent in the reactions with wild-type RNAP compared to reactions with P7-sensitive RNAP (Fig. 3a, lanes 6 and 15). In the presence of P7, the radioactivity in complex C3 disappeared, and we detected the formation of complex C4 (Fig. 3a, lane 16 and 17). As expected, this P7-induced disappearance and formation of C3 and C4, respectively, was not seen in control reactions with the ^WT^Eσ^54^ (Fig. 3a, lanes 7 and 8).

To determine whether complexes C2–C4 contain σ^54^, we repeated the EMSAs with ^32^P-labelled *nifH* probe and holoenzymes reconstituted with Alexa488-fluorophore-labelled versions of σ^54^ (σ^54^*) and analysed the gels by autoradiography and fluorescence imaging (the same reactions were split and electrophoresed using two separate gels run in the same gel tank). The fluorescence image of the gel containing reactions with wild-type RNAP revealed that the C2 complex did not contain σ^54⁎^ [Fig. 3b, (i), lanes 6 and 6′]. Since < 5 nM of σ^54⁎^ (which is the maximum amount of σ^54⁎^ that could potentially be in complex C2) is within the detection limit of our fluoroimager, we are confident that C2 is a σ^54^-free complex. As can be clearly seen in the autoradiographs and fluorescence images of gels containing both the wild-type and P7-sensitive RNAP, complex C3 is composed of the core RNAP, *nifH* probe, and σ^54⁎^, and thus, we consider this complex to be the RPc [Fig. 3b, (i and ii), compare lanes 6 and 6′]. We note that the RPc migrates at the same position as the Eσ^54⁎^ complex [Fig. 3b, (i and ii), compare lanes 6, 6′, and 9′] under our conditions. Since complex C3, that is, the RPc, is not present in reactions containing P7 [Fig. 3b, (ii), lanes 7 and 8], we conclude that P7 inhibits transcription initiation at σ^54^-dependent promoters by preventing RPc formation. Control reactions with the wild-type core RNAP, as expected, show that C3 is unaffected by the presence of P7 (Fig. 3b, lanes 7 and 8). We note the presence of a fluorescence band [originating from σ^54^*; indicated as complex CX in Fig. 3b, (ii), lanes 7′, 8′, and 10′] on the gel containing the P7-sensitive RNAP migrates at the same position as C3 [= RPc; Fig. 3b, (ii), compare lanes 6 and 6′ with 7′, 8′, and 10′], and since P7 inhibits RPc formation (see above) and the RPc and Eσ^54⁎^ complexes co-migrate at the same position under our conditions (see above), we propose that the slower migrating fluorescent band (= complex CX) seen in lanes 7′, 8′, and 10′ could be the Eσ^54⁎^ and/or Eσ^54^^⁎^–P7 complexes (see below). Importantly, we clearly observe that σ^54^ is not present in complex C4 [Fig. 3b, (ii), compare lanes 7 and 8 with 7′ and 8′].

We next conducted EMSAs with ^32^P-labelled *nifH* probe and Alexa488-fluorophore-labelled P7 (P7*) to determine if P7 is present in the various complexes seen in Fig. 3a and b. Results shown in Fig. 4a clearly indicated that P7 is present in complex C4 (compare lanes 6 and 7 with 6′ and 7′), whereas σ^54^ is not [Fig. 3b, (ii); see above]. The results also revealed that P7 was present in complex CX seen in Fig. 3b, (ii) lanes 7′ 8′, and 10′, which confirms that neither a ternary complex consisting of core RNAP, σ^54^, *nifH* probe, *ipso facto*, and the RPc, nor a quaternary complex consisting of core RNAP, σ^54^, *nifH* probe, and P7 can exist in the presence of P7, and thus, P7 inhibits transcription at σ^54^-dependent promoters by inhibiting RPc formation. However, it seems that P7 does not detectably affect the stability of the Eσ^54^ as a ternary complex consisting of RNAP, σ^54^ and P7 can clearly exist [compare Fig. 3b, (ii), lanes 9′ and 10′ and Fig. 4a, lanes 9′ and 10′]. We also note that P7 is present in complex C5, which indicates that this complex contains the core RNAP, *nifH* probe, and P7 (Fig. 3a, lane 14, and Fig. 4a, lanes 5 and 5′). Thus, it seems that although complexes C4 and C5 consist of the same three components (core RNAP, *nifH* probe, and P7), they clearly seem to assume different conformations. Put simply, the RNAP–*nifH*–probe-P7 (= C4) complex that forms as a result of P7 action during RPc formation appears to be conformationally different to the ternary RNAP–*nifH*–probe–P7 complex (= C5) that forms in the absence of any DNA. Finally, we conducted EMSAs with ^32^P-labelled *nifH* probe and P7* to determine if P7 can disrupt preformed RPc [in other words, P7 was added to the RPc (= complex C3) prior to the separation of the complexes on the native gel]. Results in Fig. 4b show that P7 can disrupt the preformed RPc (lane 4) as efficiently as when added prior to RPc formation (lanes 2 and 3) and causes the formation of complex C4 (compare lanes 2–4 with 2′–4′). Overall, the results strongly indicate that P7 prevents RPc formation and can destabilise preformed RPc. Put simply, like at σ^70^-dependent promoters [16], in the presence of the promoter DNA, P7 seems to cause the dissociation of σ^54^ from the holoenzyme, resulting in the formation of a ternary complex core RNAP–*nifH*–probe–P7 complex (= C4) and the Eσ^54^–P7 and/or Eσ^54^ complexes, but never the RPc (core RNAP-σ^54^-*nifH*-probe complex) or a complex consisting of core RNAP, σ^54^, *nifH* probe, and P7.

In summary, we conclude that both the major and major variant forms of the bacterial RNAP are inhibited by P7 by a mechanism that involves the inhibition of RPc formation. Since the functional homologue of P7 in T7 phage, Gp2, does not inhibit transcription initiation by Eσ^54^ [25], this study demonstrates for the first time that phage-encoded transcription regulators can also potentially target the major variant form of the bacterial RNAP. Although the results clearly show that P7 inhibits RPc formation by Eσ^54^, the precise mechanism underpinning this process is unknown. We propose that the binding of P7 to the β flap/β′ NTD domains could allosterically affect other parts of the RNAP and σ^54^ associated with promoter recognition and RPc formation. This view is consistent with the previous finding that a mutant form of Eσ^54^, reconstituted with a mutant variant of the core RNAP containing a deletion of the β flap-tip helix, displayed defects at several steps after holoenzyme formation *en route* to the transcriptionally proficient RPo [26]. Equally, it is possible that P7 repositions the CBD of σ^54^, which is obligatory for the docking of σ^54^ to the RNAP and makes extensive contacts to the β′ NTD and the β flap domain (= the P7 binding regions) and thereby indirectly affects promoter DNA binding by the Eσ^54^. Intriguingly, whereas P7 has no detectable effect on Eσ^70^ activity after the RPo has formed (Fig. 2c, lane 4), P7 clearly detrimentally affects the activity of the Eσ^54^–RPo to some degree (Fig. 2a, lane 5). This observation could possibly indicate conformational differences in the RPo formed by Eσ^70^ and Eσ^54^; whereas the P7 interacting regions are accessible for P7 binding in the Eσ^54^–RPo, this seems to be not the case in the Eσ^70^–RPo.

The transcriptional programme of the Xp10 phage clearly relies on the coordinated activity of both the host and Xp10 RNAPs. During early stages of infection, Xp10 relies on the *X. orzyae* Eσ^70^ because several σ^70^-dependent promoters drive the transcription of early Xp10 genes. The host RNAP becomes dispensable for the transcription of late Xp10 genes, and P7 facilitates the switching between the host and phage RNAP [27], [28]. The results presented here, although derived from using an altered version of the *E. coli* RNAP, in which the aa residues 6–9 (NLFN) of the β′ subunit are substituted with the corresponding residues from the *X. oryzae* β′ subunit (the major determinant for P7 binding), suggests that P7 can inhibit RPc formation by the major and major variant forms of the *X. oryzae* RNAP, thus suggesting that the Xp10 transcription programme might require or involves the inactivation of the host transcription machinery containing σ^54^. The use of σ^54^ by phages for the execution of their transcriptional programme, although rare, is not unprecedented, since the development of the *Pseudomonas aeruginosa* phage YuA is strictly dependent on the host σ^54^ factor [29].

Our results further indicate that regardless of the nature of the σ factor–β flap–β′ NTD interface, P7 is able to indiscriminately prevent the productive and efficient engagement of the RNAP with the promoter and thereby underscores the significance of the β flap/β′ NTD domains for bacterial RNAP function and regulation. Since the RNAP is a proven antibacterial target, the σ factor–β flap–β′ NTD interface is potentially an Achilles' heel in the bacterial RNAP for intervention by small molecules to inhibit bacterial transcription.

## Acknowledgements

This work was funded by project grants from the BBSRC and the Wellcome Trust. S.W. and S.M. are recipients of Investigator Awards from the Wellcome Trust.

## Figures and Tables

**Fig. 1:**
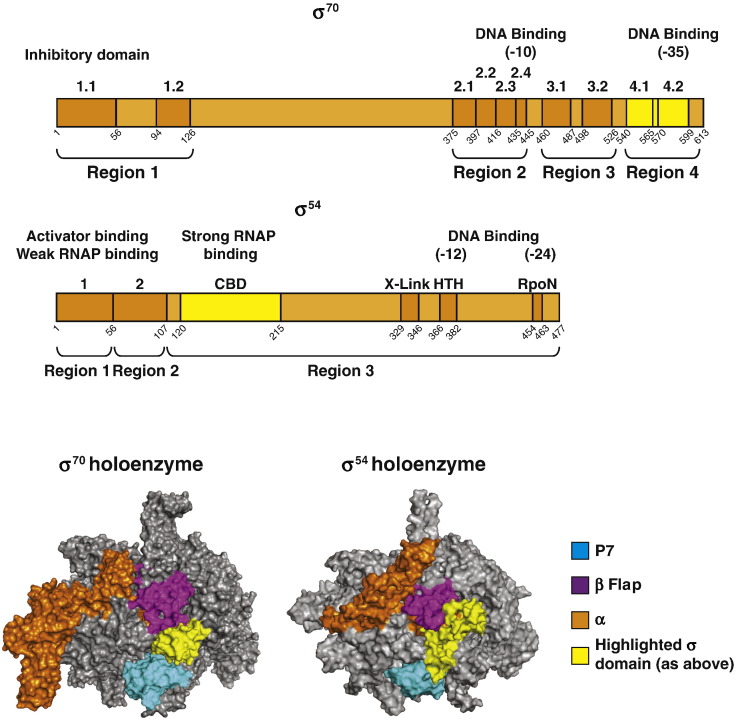
Structural models of P7 bound to the major and major variant forms of the bacterial RNAP. (above) Schematic representation of the domain organisations of σ^70^ and σ^54^. In yellow are domains that are proximal to P7 interacting surfaces on the core RNAP (see text for details). (below) Surface representation of the structural models of P7 bound to the *E. coli* σ^70^ and σ^54^ holoenzymes (derived from PDB 4YG2 and PDB 5BYH, respectively [7], [21]). The β flap, P7, and σ factors are coloured as indicated in the key.

**Fig. 2:**
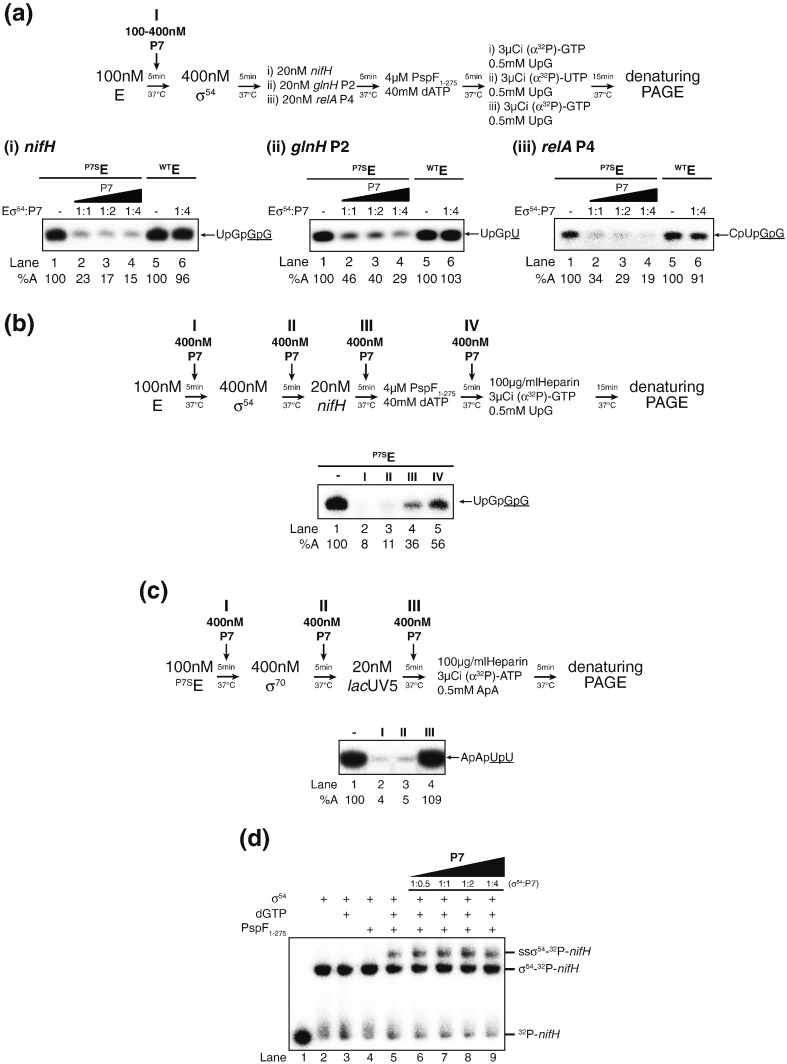
P7 inhibits transcription initiation by the σ^54^-containing RNAP. (a) An autoradiograph of a 20% (wt/vol) denaturing gel showing the synthesis of the transcript by ^P7S^Eσ^54^ (lanes 1–5) or ^WT^Eσ^54^ (lanes 7 and 8) in the absence and presence of increasing amount of P7 from the following σ^54^-dependent promoters: (i) *nifH* (transcript = UpGpGpG), (ii) *glnH* P2 (transcript = UpGpU), and (iii) *relA* P4 (transcript = CpUpGpG). All transcripts are indicated by an arrow where the underlined nucleotides are 32P labelled. P7 was added to the reactions prior to holoenzyme formation as is indicated in the schematic. All data obtained in at least three independent experiments fell within 5% of the relative %A value shown. (b) An autoradiograph of a 20% (wt/vol) denaturing gel showing the synthesis of the transcript UpGpGpG (indicated by the arrow, where the underlined nucleotides are 32P labelled) from the *nifH* promoter by ^P7S^Eσ^54^ in the absence and presence of P7. P7 was added to the reaction at different stages, indicated by the numerals I–IV: I = prior to holoenzyme formation, II = prior to RPc formation, III = prior to RPo formation, and IV = after RPo formation. The percentage of synthesised UpGpGpG (%A) indicates the activity of the RNAP in the presence of P7 compared to reactions with no P7 present (lane 1). All data obtained in at least three independent experiments fell within 5% of the relative %A value shown. (c) An autoradiograph of a 20% (wt/vol) denaturing gel showing the synthesis of the transcript ApApUpU (indicated by the arrow, where the underlined nucleotides are ^32^P labelled) from the *lac*UV5 promoter by ^P7S^Eσ^70^, where P7 was added to the reaction at different stages, indicated by the numerals I–III: I = prior to holoenzyme formation, II = prior to RPc formation, and III = after RPo formation. The percentage of synthesised ApApUpU (%A) indicates the activity of the RNAP in the presence of P7 compared to reactions with no P7 present (lane 1). In at least three independent experiments, all data obtained fell within 5% of the %A value shown. (d) Autoradiograph of 4.5% (wt/vol) native polyacrylamide gel showing results from an EMSA to determine whether P7 affects the formation of an activation-dependent (here using phage shock protein F_1–275_ and 4 mM dGTP), remodelled σ^54^–DNA complex (indicated as super-shifted or ssσ^54^–^32^P–*nifH*). In lanes 5–9, the presence of increasing amounts of P7 had no detectable effect on ssσ^54^–^32^P–*nifH* formation. (a–d) In the schematic above, autoradiograph images indicate the concentration of reaction components, the point in time they were added to the reactions and the incubation times; the migration positions of protein and DNA components in each lane are indicated, and the assays were conducted essentially as previously described. The experiments in (a–d) were conducted as described in Refs [26], [30], [24], respectively.

**Fig. 3:**
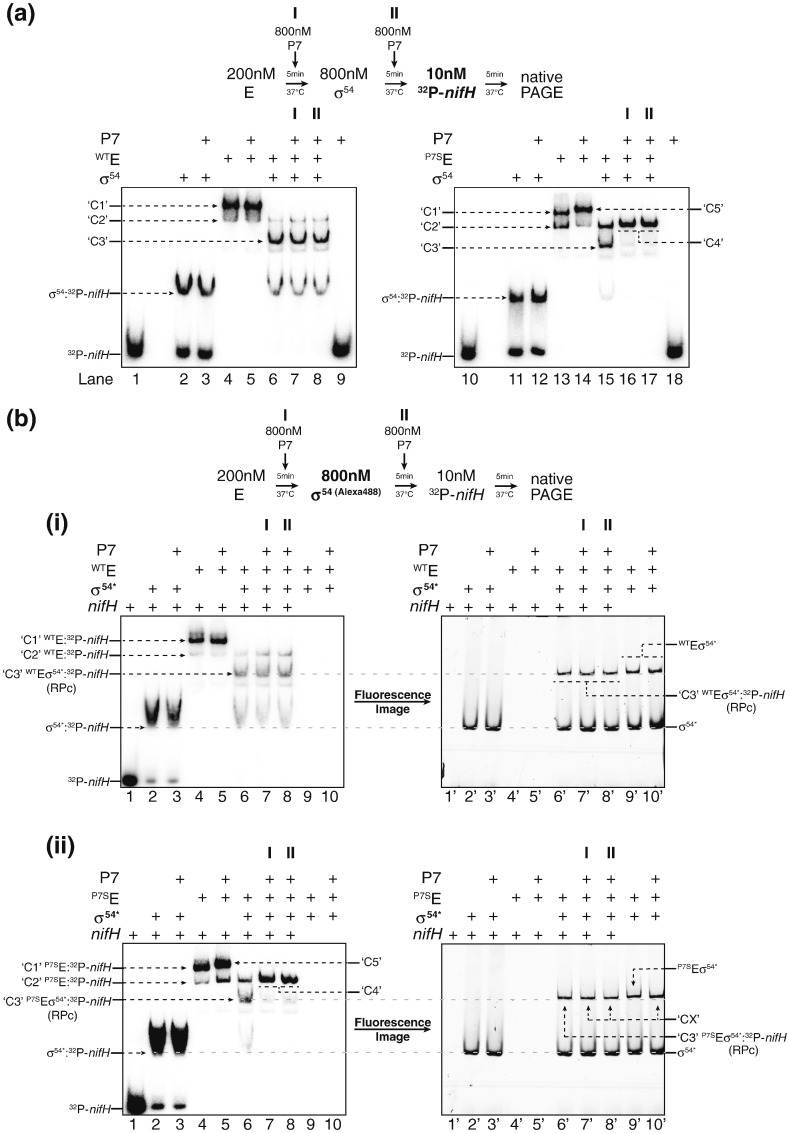
P7 prevents RPc formation by the σ^54^-containing RNAP. (a) Autoradiograph of a 4.5% (wt/vol) native polyacrylamide gel showing results from EMSA experiment with ^32^P-labelled *nifH* promoter probe to demonstrate that P7 inhibits RPc formation by the σ^54^ holoenzyme conducted as previously described [16], [30]. The components present in each lane are indicated above each image of the gel, and the schematic indicates the concentration of reaction components, time of addition, and incubation time. (b) As in (a), but the assays were conducted with Alexa488-labelled σ^54^ (σ^54^*) to determine the presence or absence of σ^54^ in the different complexes detected in (a) by fluorescence imaging. The Alexa488-labelled version of σ^54^ was prepared as described in Ref. [31]. In (a and b), the migration positions of the different protein–protein and protein–DNA complexes are indicated (see text for details). We note that we could not clearly distinguish the free σ^54^* and σ^54^*–^32^P–*nifH* complex in the gels shown on the right in Fig. 3b. We explain this by suggesting that the excess of free σ^54^* (800 nM) may mask the amount of σ^54^*–^32^P–*nifH* complexes formed (maximum of 10 nM) under our experimental conditions. The gels analysed by radiography were dried prior to exposure to the phosphorimaging plate, whilst gels analysed by fluorescence were not dried.

**Fig. 4:**
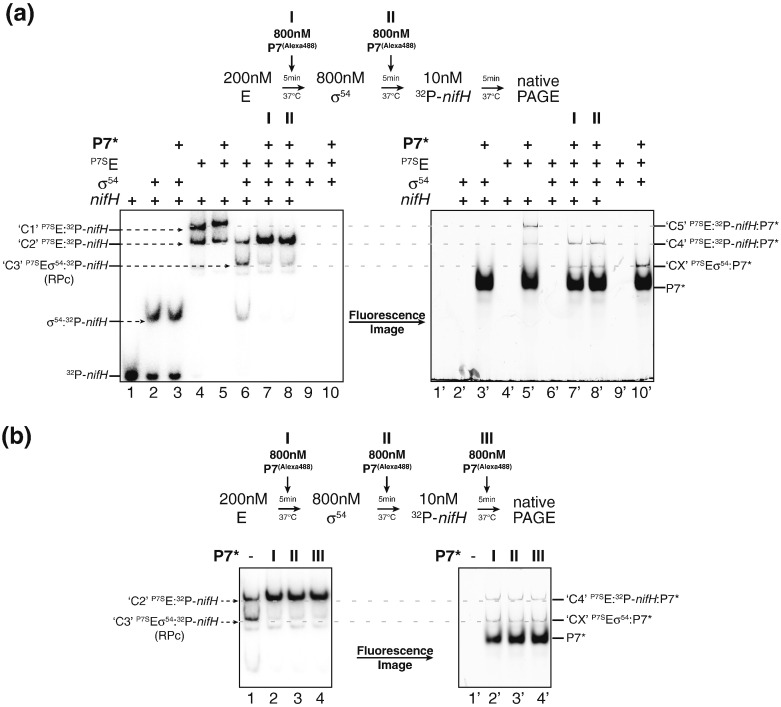
P7 inhibits RPc formation by the σ^54^-containing RNAP but does not fully dissociate the σ^54^–RNAP holoenzyme. Autoradiograph and fluorescent image of a 4.5% (wt/vol) native polyacrylamide gel showing results from EMSA experiment with ^32^P-labelled *nifH* promoter probe to demonstrate that P7 inhibits RPc formation by the σ^54^ holoenzyme conducted as previously described [16], [30]. (a) and (b) are essentially completed as in Fig. 3a, but the assays were conducted with Alexa488-labelled P7 (P7*) to determine the presence or absence of P7 in the different complexes detected in Fig. 3a. The Alexa488-labelled version of P7 was prepared as described in Ref. [31]. The components present in each lane are indicated above each image of the gel, and the schematic indicates the concentration of reaction components, time of addition, and incubation time. In (a and b), the migration positions of the different protein–protein and protein–DNA complexes are indicated (see text for details). Note that the gels analysed by radiography were dried prior to exposure to the phosphorimaging plate, whilst gels analysed by fluorescence were not dried.
